# Risk factors for short- and long-term complications after groin surgery in vulvar cancer

**DOI:** 10.1038/bjc.2011.407

**Published:** 2011-10-04

**Authors:** F Hinten, L C G van den Einden, J C M Hendriks, A G J van der Zee, J Bulten, L F A G Massuger, H P van de Nieuwenhof, J A de Hullu

**Affiliations:** 1 Department of Obstetrics and Gynaecology, Radboud University Nijmegen Medical Centre, PO Box 9101, 6500 HB Nijmegen, The Netherlands; 2 Department of Statistics, Radboud University Nijmegen Medical Centre, Nijmegen, The Netherlands; 3 Department of Obstetrics and Gynaecology, University Medical Centre Groningen, Groningen, The Netherlands;; 4 Department of Pathology, Radboud University Nijmegen Medical Centre, Nijmegen, The Netherlands

**Keywords:** vulvar cancer, postoperative complications, morbidity, surgery

## Abstract

**Background:**

The cornerstone of treatment in early-stage squamous cell carcinoma (SCC) of the vulva is surgery, predominantly consisting of wide local excision with elective uni- or bi-lateral inguinofemoral lymphadenectomy. This strategy is associated with a good prognosis, but also with impressive treatment-related morbidity. The aim of this study was to determine risk factors for the short-term (wound breakdown, infection and lymphocele) and long-term (lymphoedema and cellulitis/erysipelas) complications after groin surgery as part of the treatment of vulvar SCC.

**Methods:**

Between January 1988 and June 2009, 164 consecutive patients underwent an inguinofemoral lymphadenectomy as part of their surgical treatment for vulvar SCC at the Department of Gynaecologic Oncology at the Radboud University Nijmegen Medical Centre. The clinical and histopathological data were retrospectively analysed.

**Results:**

Multivariate analysis showed that older age, diabetes, ‘en bloc’ surgery and higher drain production on the last day of drain *in situ* gave a higher risk of developing short-term complications. Younger age and lymphocele gave higher risk of developing long-term complications. Higher number of lymph nodes dissected seems to protect against developing any long-term complications.

**Conclusion:**

Our analysis shows that patient characteristics, extension of surgery and postoperative management influence short- and/or long-term complications after inguinofemoral lymphadenectomy in vulvar SCC patients. Further research of postoperative management is necessary to analyse possibilities to decrease the complication rate of inguinofemoral lymphadenectomy; although the sentinel lymph node procedure appears to be a promising technique, in ∼50% of the patients an inguinofemoral lymphadenectomy is still indicated.

Vulvar squamous cell carcinoma (SCC) is a rare disease and accounts for ∼3–5% of all female genital malignancies (Hacker, 2005). The incidence is ∼1–2 per 100 000 (van de Nieuwenhof *et al*, 2009). The majority of the patients with vulvar SCC have early-stage disease: a cT1 (<2 cm) or cT2 (>2 cm) tumour without suspicious inguinal lymph nodes. The standard treatment of early-stage SCC of the vulva consists of wide local excision (WLE) of the tumour combined with an inguinofemoral lymphadenectomy (removal of all superficial lymph nodes and the medial femoral lymph nodes) (Levenback *et al*, 1996; de Hullu *et al*, 2004). The inguinofemoral lymphadenectomy has significant short- and long-term complications, which are a major concern for both patients and clinicians. Wound breakdown, wound infection, formation of lymphoceles, development of lymphoedema and cellulitis/erysipelas are the most documented complications, occurring in up to 85% of the patients (Podratz *et al*, 1983; Gaarenstroom *et al*, 2003).

Only 25–35% of patients with early-stage disease will have lymph node metastases (Hacker *et al*, 1981; Burger *et al*, 1995; Bell *et al*, 2000; Katz *et al*, 2003). There are no noninvasive techniques such as palpation, ultrasound, CT, PET and MRI available with a high enough negative predictive value to safely omit inguinofemoral lymphadenectomy in a selection of patients (Oonk *et al*, 2006). This urged the introduction of the sentinel lymph node (SLN) procedure in vulvar SCC. After excellent results in different accuracy studies (Ansink *et al*, 1999; De Cicco *et al*, 2000; de Hullu *et al*, 2000; Levenback *et al*, 2001; Sliutz *et al*, 2002; Moore *et al*, 2003), van der Zee *et al* (2008) showed in the ‘Groningen International Study on Sentinel nodes in Vulvar cancer I’ (GROINSS-V I) with the combined technique that in early-stage vulvar SCC patients with a negative SLN, the groin recurrence rate is low, survival is excellent and the treatment-related morbidity is minimal.

Despite the excellent outcomes of the SLN procedure, only patients with small (<4 cm) unifocal tumours are eligible for this technique. Therefore, in ∼50% of the patients, there is still an indication for inguinofemoral lymphadenectomy. The modifications of the past decades have been introduced to decrease morbidity without compromising prognosis. ‘En bloc’ surgery has been replaced by the triple incision technique (de Hullu *et al*, 2002). Performing a superficial lymphadenectomy alone gives a decrease in survival (Stehman *et al*, 1992a; Burke *et al*, 1995), and hence at least the lymph nodes medial of the femoral vessels should be removed. In the literature, sparing of the saphenous vein does not reduce lymphoedema in all studies (Podratz *et al*, 1983; Zhang *et al*, 2000; Rouzier *et al*, 2003). Sartorius transposition did not decrease the morbidity (Rouzier *et al*, 2003; Judson *et al*, 2004).

The direct postoperative management for patients with vulvar SCC has not been described extensively. Gould *et al* (2001) showed that prophylactic antibiotics and duration of drains *in situ* were no predictors for the development of wound infection and late complications (lymphoedema and cellulitis). The drains were removed when the output was <30 ml per day. Gaarenstroom *et al* (2003) described that the drains were removed when the fluid production was <50 ml per day after at least 5 days. However, the reason for this specific duration was not based on study results. In breast cancer, the postoperative management after axillary lymphadenectomy has been studied in more detail. There is no clear evidence that the use of a drain after axillary surgery reduces the incidence of lymphocele formation (Zavotsky *et al*, 1998; Talbot and Magarey, 2002; Soon *et al*, 2005). The studies in breast cancer that compared early with late drain removal (Inwang *et al*, 1991; Gupta *et al*, 2001; Dalberg *et al*, 2004) concluded that early drain removal was safe, but that the incidence of lymphoceles was higher in this group.

The aim of this study is to investigate the influence of patients’ characteristics, extension of surgery and postoperative management on the short- and long-term complication rate after inguinofemoral lymphadenectomy in patients with SCC of the vulva.

## Patients and methods

### Patients

Data of 283 consecutive patients with vulvar SCC who were treated at the Department of Gynaecologic Oncology at the Radboud University Nijmegen Medical Centre (RUNMC) between 1 January 1988 and 30 June 2009 were retrieved from medical files. A total of 78 patients were excluded from the current analysis because their groins were not treated surgically (*n*=8), the primary treatment took place in another medical centre (*n*=21), no groin surgery was performed at primary treatment (*n*=36), only superficial inguinal lymphadenectomy was performed (*n*=4) only debulking of lymph node metastases was performed (*n*=2) or posterior exenteration was performed (*n*=3). Four patients were excluded because their medical files could not be retrieved. In 205 patients groin surgery was performed; 41 patients only underwent SLN procedure and were excluded. In 2001, the SLN procedure (unilateral or bilateral) was introduced in the RUNMC initially in an accuracy study (followed by lymphadenectomy) that preceded the GROINSS V-studies by van der Zee *et al* (2008). Data of 164 patients were available for further analysis in this study. Local surgery consisted of a WLE or radical vulvectomy. From 1988 to 1993, standard local treatment consisted of a radical vulvectomy. After 1993, the WLE was introduced; it was carried out when the tumour was clinically resectable with a macroscopically measured normal tissue margin of 1–2 cm despite the tumour diameter. After the introduction of the WLE, radical vulvectomy was only considered in patients with multifocal tumours and in case of an abnormal remainder of the vulva with complaints. Groin surgery consisted of ‘en bloc’ inguinofemoral lymphadenectomy from 1988 to 1993. In 1993, the triple incision technique was introduced (de Hullu *et al*, 2002): when the medial margin of the tumour was >1 cm from the midline, unilateral or otherwise a bilateral inguinofemoral lymphadenectomy was performed. A total of 62% of our patients underwent inguinofemoral lymphadenectomy through triple incision technique after 1993 *vs* 17% before 1993. It took some time until the triple incision technique was fully integrated in our Gynaecologic Oncology centre. The inguinofemoral lymphadenectomy contained resection of superficial lymph nodes as well as deep femoral nodes. For the resection of inguinal lymph nodes, the fatty tissue beneath the subcutaneous tissue down to the fascia lata was removed. The saphenous vein was spared when possible. After splitting the fascia lata, the fatty tissue medial to the femoral vessels within the opening of the fossa ovalis was resected to perform femoral lymphadenectomy. The lateral part of the fascia lata was spared and no sartorius transposition was performed.

### Data

All data were retrospectively collected from a database and the patient charts. Parameters extracted were: patients’ characteristics (age, diabetes, peripheral vascular disease, body mass index (BMI) and continuation of antibiotics), type of surgery (‘en bloc’ approach or triple incision technique, unilateral or bilateral inguinofemoral lymphadenectomy, the ligation of the saphenous vein, number of removed lymph nodes, presence or absence of lymph node metastases and adjuvant radiotherapy) and postoperative management (drain management). In the RUNMC, all patients received standard antibiotics during surgery: Cefazoline 1000 mg and Metronidazol 500 mg; in some individual patients, the treatment with antibiotics extended for some additional days. ‘Antibiotics’ in our study was defined as the continuation of antibiotics after surgery. Patients who underwent an inguinofemoral lymphadenectomy received high-vacuum Redon drains (775 mm Hg (0.9 bar) negative pressure) in the groins postoperatively. In general, the drains were *in situ* for 5 days and these were removed when the production was decreasing and under 50–100 ml per day. ‘Duration of the drains in the groins’ was defined as the time between operation and the day the drains were removed. The ‘fluid production’ was measured per day. Prescription of elastic stockings was a standard procedure in patients who underwent inguinofemoral lymphadenectomy. ‘Hospitalisation time’ was defined as the day of operation (day 0) and the number of postoperative days in the hospital. The influence of adjuvant radiotherapy was only assessed for the long-term complications.

Definitions and the frequencies of the short- and long-term complications after inguinofemoral lymphadenectomy are shown in Table 1. In total, 137 patients (84%) suffered from a complication of any kind after inguinofemoral lymphadenectomy. We also assessed the frequency of any of the short-term complications and any of the long-term complications. Standard follow-up was every 3 months in the first 2 years; from the third to the fifth year, it was twice a year and yearly thereafter.

### Statistical methods

All events were described per groin, but analysis of complication rate per groin might overrate the influence of patient characteristics, because these were doubled in case of a bilateral lymphadenectomy. In patients who underwent bilateral lymphadenectomy, we randomly analysed the right or the left groin in order to minimise bias. We started at the top of the database and took the right groin in the first patient and the left groin in the second and so on without knowing in which groin the complications occurred.

Variables eligible for entry were analysed using SPSS software (version 16.0.01 for Windows, SPSS, Chicago, IL, USA). Univariate logistic regression was used to assess the risk of patients’ characteristics, type of surgery and postoperative management on the short-term complications and long-term complications, respectively any of the short-term complications and the long-term complications as the single type complications. The odds ratios with the 95% confidence interval (CI) are presented. Multivariate logistic regression with forward selection procedure was used to identify those variables that independently contributed to the risk of short-term complications and long-term complications (statistically significant variables from univariate logistic regression). After entry the adjusted odds ratios with 95% CI of the final model are presented. A *P*-value of <0.05 was considered statistically significant.

An IRB approval was not necessary for this retrospective study.

## Results

**Figure 1:**
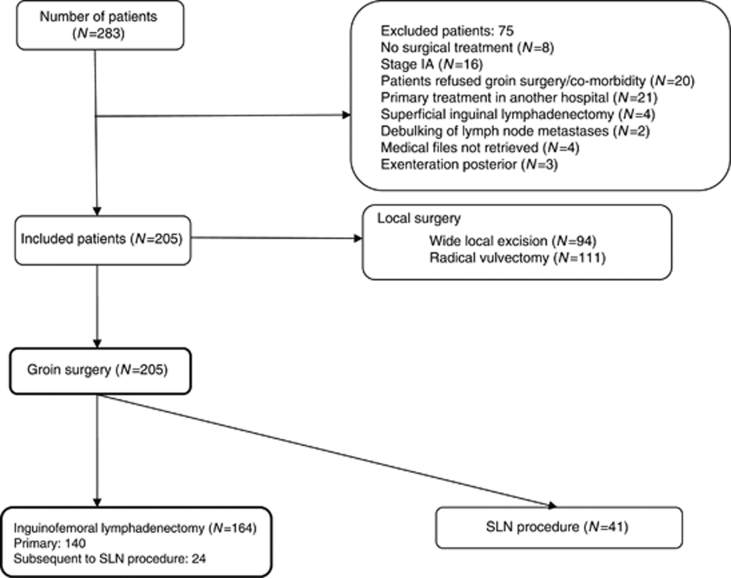
Inclusion chart.

Of all patients who underwent inguinofemoral lymphadenectomy for primary SCC of the vulva (*n*=164), 140 patients underwent primary inguinofemoral lymphadenectomy, whereas 24 patients underwent inguinofemoral lymphadenectomy subsequent to SLN procedure during the learning curve (with standard inguinofemoral lymphadenectomy after SLN procedure) or because of positive SLN(s) in GROINSS-V I (van der Zee *et al*, 2008). In 301 groins of 164 patients, an inguinofemoral lymphadenectomy was performed (27 patients only unilateral), of which 73 patients underwent surgery through the ‘en bloc’ approach. Figure 1 shows a flowchart of patients with SCC who underwent groin surgery. Table 2 shows the features of the research population.

The SLN procedure was not yet introduced in our department before 2001. In retrospect, ∼50% of the patients in our study population were not or would not have been eligible for a SLN procedure because the tumour was >4 cm and/or multifocal. The details of use (duration and results) of the stockings were not well documented in the medical charts, and hence this item was excluded from the analysis.

Risk factors for short-term complications and long-term complications were assessed with univariate analysis (Tables 3 and 4).

Using multivariate analysis, ‘en bloc’ surgery (odds ratio 2.72, 95% CI 1.16–6.37) and older age (odds ratio 1.06, 95% CI 1.02–1.10) were both independent risk factors for developing wound breakdown. ‘En bloc’ surgery (odds 2.66, 95% CI 1.15–6.15) and higher drain production on the last day the drain was *in situ* (odds ratio 1.05, 95% CI 1.00–1.09) were the only independent risk factors for wound infection. Higher drain production on the last day the drain was *in situ* (odds ratio 1.05, 95% CI 1.01–1.10) was a risk factor for developing lymphocele. Diabetes (odds ratio 4.10, 95% CI 1.04–16.05) and higher drain production on the last day the drain was *in situ* (odds ratio 1.11, 95% CI 1.04–1.19) were risk factors for developing any of the short-term complications. Younger age was the only independent risk factor for developing lymphoedema (odds ratio 0.95, 95% CI 0.93–0.98). The independent risk factors for cellulitis/erysipelas were younger age (odds ratio 0.96, 95% CI 0.93–0.98) and lymphocele (odds ratio 3.28, 95% CI 1.50–7.19). Higher number of lymph nodes dissected seems to protect against developing any long-term complications (odds ratio 0.92, 95% CI 0.84–1.00) and younger age was a risk factor (odds ratio 0.94, 95% CI 0.92–0.97; Table 5).

## Discussion

In this study we found different risk factors for the short- and long-term complications after inguinofemoral lymphadenectomy as part of the standard treatment for primary vulvar SCC. Older age, diabetes, ‘en bloc’ surgery and higher drain production on the last day the drain was *in situ* were significant risk factors for short-term complications. Younger age and lymphocele gave higher risk of developing long-term complications.

We found that older age was associated with higher risk for wound breakdown. This can be explained by the deterioration of wound healing with age. On the other hand, younger age was correlated with the long-term complications lymphoedema and cellulitis/erysipelas. One should realise that younger women are more active and might be more limited in their daily activities by possible lymphoedema; older people may experience more restrictions from other diseases, such as cardiac problems resulting in lymphoedema. Our study showed that diabetes was associated with wound breakdown and any short-term complication. It is well known that diabetes mellitus is associated with wound healing problems in many surgical disciplines (Trussell *et al*, 2008; Chen *et al*, 2009; McConnell *et al*, 2009; Ogihara *et al*, 2009). Therefore, the glucose levels in patients with diabetes should be regulated strictly to diminish the influence of diabetes on the short-term complications.

In our study the ‘en bloc’ approach was the only surgical technique-related risk factor found. In 1993, the triple incision technique was introduced in the RUNMC. In our study we found a decrease in complication rate after 1993, especially in the short-term complication rate (76.2% before 1993 and 55.7% after 1993). We did expect to find this result, as our study showed, as expected in literature (Hacker *et al*, 1981; Podratz *et al*, 1983; Lin *et al*, 1992), the ‘en bloc’ approach to be a risk factor for both wound breakdown and wound infection. This can also be explained by the higher rate of triple incisions performed after 1993 compared with before 1993 (61.7% *vs* 17.4%). Nowadays, ‘en bloc’ surgery is only performed in patients with large suspicious inguinofemoral lymph nodes to prevent skin bridge and groin recurrences. We hypothesised that a higher total amount of dissected lymph nodes during surgery would impose a risk for lymphoedema, which was based on the idea that less lymph nodes may drain less lymph fluid. The mean number of nodes dissected in our study was 9.45 nodes per groin, and a higher amount of nodes dissected as a risk factor for lymphoedema was not confirmed in this study. On the contrary, a higher amount seemed to protect against developing any long-term complications. Besides, only in cellulitis/erysipelas a cutoff point was recognised, namely 10 lymph nodes (>10 lymph nodes dissected posed protection). We did not have an explanation for this finding. Courtney-Brooks *et al* (2010) showed that removal of >10 lymph nodes might be associated with better survival in FIGO stage III patients. The prognostic impact of the number of lymph nodes dissected remains unclear. It is advised to remove between 6 and 8 lymph nodes per groin (Butler *et al*, 2010; Woelber *et al*, 2011), but variations in anatomy and other factors make node counting an unreliable measure of surgical quality (Stehman *et al*, 2009).

In melanoma of the lower extremities, patients also undergo lymphadenectomy, in most cases combined with pelvic lymphadenectomy. There are comparable complication rates described as in vulvar cancer patients: wound breakdown 3–26%, wound infection 9–30%, lymphocele 5–46% and lymphoedema 20–64% (Baas *et al*, 1992; Beitsch and Balch, 1992; Karakousis and Driscoll, 1994; Lawton *et al*, 2002; Serpell *et al*, 2003; de Vries *et al*, 2006). Apparently, morbidity after inguinofemoral lymphadenectomy is impressive despite the type of the primary tumour.

The use of drains after inguinofemoral lymphadenectomy is generally accepted worldwide and therefore used in our gynaecologic oncology department. There are no standardised protocols for the duration of drainage, but in most cases the drains are left *in situ* for at least 5 days; the postoperative management at the RUNMC is to remove the drains when the production has decreased under 50–100 ml per day. Only two retrospective studies on postoperative management in vulvar SCC report a postoperative protocol on drain management; either remove the drain when the output was <30 ml per day or when the fluid production was <50 ml per day after at least 5 days (Gould *et al*, 2001; Gaarenstroom *et al*, 2003). Both studies showed, in accordance with our results, that duration of the drain *in situ* had no influence on the short- and long-term complications after inguinofemoral lymphadenectomy. There is limited literature on drain management in patients after inguinofemoral lymphadenectomy for vulvar SCC, probably because of the low incidence of vulvar SCC and/or the focus on improving quality of life by the SLN procedure.

In contrast with the groin, drain management in the axilla after breast cancer treatment has been extensively studied; most surgeons remove the drain when the drainage volume is <20–50 ml in the preceding 24 h and this may take up to 10 days (Tadych and Donegan, 1987; Yii *et al*, 1995; Bundred *et al*, 1998; Kopelman *et al*, 1999; Woodworth *et al*, 2000). Barwell *et al* (1997) showed that patients who developed a lymphocele after breast cancer surgery had a higher mean total drain volume (480 ml) than patients who did not develop a lymphocele (240 ml). We found that the total volume of fluid drained from the groin was ∼1.5 times higher, without a significant difference between the patients who did and did not develop a lymphocele. An explanation may be that the lymph nodes of the groin have to drain more lymph fluid from the lower extremities than the lymph nodes in the axilla from the upper extremities.

Our study showed that a higher drain production on the last day that the drain was *in situ* was associated with an increased risk for lymphocele formation. An explanation for this result is that after removal of the drain, stasis of lymph fluid takes place, which gives rise to lymphoceles. Our study is limited by a small group of patients with known drain production on the last day. These results confirm our hypothesis that more the fluid drained on the last day, the higher the incidence of lymphoceles would be as shown in the studies on breast cancer. In the literature on breast cancer surgery, the amount of postoperative fluid drainage has been found to be significantly influenced by the degree of negative pressure in the drain. The hypothesis is that a high negative suction pressure in the drain may prevent the leaking lymphatics and blood vessels from sealing off, thus leading to prolonged drainage (van Heurn and Brink, 1995; Kopelman *et al*, 1999; O’Hea *et al*, 1999; Chintamani *et al*, 2005). In vulvar SCC, high-vacuum drains are used, but none of the studies defined the amount of negative pressure applied in the drains. There is one prospective study that compared two types of drains, the Blake and the Jackson-Pratt drain. This study showed an increased incidence of overall complication rate associated with the Blake drain (Carlson *et al*, 2008). These findings show that there is a need for further studies to investigate drain management after inguinofemoral lymphadenectomy.

Compared with a full lymphadenectomy, the SLN procedure has been shown to significantly reduce postoperative morbidity in the GROINSS-V I study (van der Zee *et al*, 2008). Our data revealed a number of patients who are not eligible for the SLN procedure because of the size of the tumour or multifocality. These patients would still require an inguinofemoral lymphadenectomy with the associated morbidity. Despite the application of the SLN procedure, the complication rate remains high compared with the rates described in the literature (Table 6). This may be explained by the different definitions for complications used in the literature. Apparently, it is difficult to prevent short- and long-term complications other than omitting lymphadenectomy. In the past years, different methods such as ligation of VSM (Podratz *et al*, 1983; Zhang *et al*, 2000; Rouzier *et al*, 2003), sartorius muscle transposition (Rouzier *et al*, 2003; Judson *et al*, 2004) and sealing with VH fibrin sealant (Carlson *et al*, 2008) have been adopted with the attempt to decrease the complication rate, but none of these methods decreased the complication rate after inguinofemoral lymphadenectomy. Hopefully, GROINSS-V II will show that radiotherapy is an attractive and safe alternative for inguinofemoral lymphadenectomy in a substantial number of patients with a positive SLN. A few other treatment options have been described in the literature on vulvar cancer. Primary radiotherapy could be able to replace inguinofemoral lymphadenectomy in patients without suspicious groins. Three studies showed that primary radiotherapy to the groin results in less morbidity but also in a higher number of groin recurrences compared with surgery (Stehman *et al*, 1992b; Manavi *et al*, 1997; Perez *et al*, 1998). It has been suggested to remove only the bulky lymph nodes before radiotherapy. A study by Hyde *et al* (2007) showed that the survival is not compromised by only resecting the bulky nodes; however, because of the small study group retrospective nature of the study, a randomised prospective study is recommended. Primary neoadjuvant chemoradiation may be an option in patients with nonresectable tumours to reduce tumour volume, achieve resectability and reduce the extent of surgery. However operability was achieved in 63–92% of cases, surgical interventions after chemoradiation are associated with high postoperative morbidity (van Doorn *et al*, 2006). Chemotherapy as a single treatment modality is not common. The data available for any of the applied chemotherapeutic regimens are not sufficient to recommend routine application (Wagenaar *et al*, 2001; Cormio *et al*, 2009; Witteveen *et al*, 2009). Only primary radiotherapy decreases the postoperative morbidity, but with compromising the prognosis. We should keep in mind that a groin recurrence is nearly always fatal. Hence, we recommend treating all patients with vulvar cancer optimally; WLE and groin surgery unless patients are unfit to undergo surgery. Furthermore, research should focus on development of tailor-made postoperative therapy such as appropriate postoperative drain management and possibly lymph drainage therapy for the individual patient who still needs the inguinofemoral lymphadenectomy to survive vulvar SCC.

In conclusion, age, diabetes, ‘en bloc’ surgery and higher drain production on the last day the drain was *in situ* are risk factors for the development of short- and long-term complications. The postoperative drain management is the only factor that urges us to further studies to find the optimal postoperative protocol. Considering the rarity of SCC of the vulva, this study should preferably be a randomised multicentre study in patients who undergo standardised bilateral inguinofemoral lymphadenectomy. Two different policies with respect to postoperative management may be studied in both groins of the same patient to exclude bias by patient-related factors.

## Figures and Tables

**Table 1:** Overview of short- and long-term complications of the groin after inguinofemoral lymphadenectomy (*N*=164)

	***n/N (%)***	
*Short term (<4 weeks)*		
Wound breakdown	30/160 (18.8)	Disrupted groin wound(s) >2 cm
Wound infection	46/161 (28.6)	Purulent exudates and/or positive culture and erythema, oedema and localised pain
Lymphocele	47/161 (29.2)	Clinically obvious and/or confirmed by puncture or ultrasound scan
Any short-term complication	94/161 (57.3)	One or more of the three short-term complications
		
*Long term (>4 weeks)*		
Lymphoedema	78/160 (48.8)	Elastic bandages or other forms of lymph drainage were required
Cellulitis/erysipelas	54/160 (33.8)	Erythematous and scalding skin with/without positive culture for *Streptococcus* and T >39 °C
Any long-term complication	102/160 (63.8)	One or more of the two long-term complications

Abbreviations: *N*=patients with valid observation; *n*=number of patients with a complication.

**Table 2:** Features of the research population with median values and percentages

**Variables**	***N***	**Median (range)**	***n* (%)**
*Patient characteristics*			
Age (years)	164	71 (31–92)	—
Diabetes	164	—	19 (11.6)
Peripheral vascular disease	164	—	8 (4.9)
BMI (kg m^–2^)	155	26.6 (17.6–48.4)	—
Continuation of antibiotics	162	—	99 (61.1)
			
*Surgical technique*			
Bilateral IFL	164	—	137 (83.5)
En bloc	164	—	73 (44.5)
Ligation saphenous vein	160	—	32 (20.0)
Total nodes dissected (no. of nodes)	164	9 (0–25)	—
Total positive nodes (no. of nodes)	164	0 (0–7)	—
Extracapsular growth (no. of nodes)	164	0 (0–7)	—
			
*Postoperative management*			
Duration drain (days)	149	8 (0–27)	—
Drain production last day (ml)	122	40.0 (0–860)	—
Total drain production (ml)	145	630 (20–7540)	—
Adjuvant radiotherapy	163	—	40 (24.5)
			
Hospital stay (days)	164	15 (4–60)	—
Follow-up (months)	164	50.3 (0.1–215)	—
			
*FIGO stage (new)*	164	—	
IB			78 (47.6)
II			8 (4.9)
IIIA			24 (14.6)
IIIB			11 (6.7)
IIIC			29 (17.7)
IIIA/B			9 (5.5)
IVA			4 (2.4)
IVB			1 (0.6)

Abbreviations: BMI=body mass index; FIGO=International Federation of Gynaecology and Obstetrics; *N*=number of patients with valid observation; *n*=number of patients with specific feature; IFL=inguinofemoral lymphadenectomy; —=not applicable.

**Table 3:** Odds ratios with 95% confidence interval of patient characteristics, surgery and postoperative management variables for short-term complications using univariate logistic regression

	**Wound breakdown**	**Wound infection**	**Lymphocele**	**Any short-term complication**				
**Variables**	***N***	**OR (95% CI)**	***N***	**OR (95% CI)**	***N***	**OR (95% CI)**	***N***	**OR (95% CI)**
Age (years)	160	1.06 (1.02–1.10)^*^	161	1.01 (0.98–1.03)	161	1.01 (0.99–1.04)	160	1.03 (1.01–1.06)^*^
								
*Diabetes*								
Yes	19	2.99 (1.07–8.14)^*^	19	1.99 (0.74–5.32)	19	0.85 (0.29–2.51)	19	4.31 (1.20–5.45)^*^
No	141	1.00 (Ref)	142	1.00 (Ref)	142	1.00 (Ref)	141	1.00 (Ref)
								
*Peripheral vascular disease*								
Yes	8	2.78 (0.63–12.33)	8	1.54 (0.35–6.70)	8	2.56 (0.61–10.69)	8	1.24 (0.00–)
No	152	1.00 (Ref)	153	1.00 (Ref)	153	1.00 (Ref)	152	1.00 (Ref)
								
BMI (in kg m^–2^)	153	1.04 (0.96–1.13)	153	1.05 (0.98–1.13)	153	0.96 (0.89–1.04)	153	1.07 (1.00–1.15)
								
*Continuation of antibiotics*								
Yes	97	1.98 (0.82–4.78)	97	1.13 (0.56–2.29)	97	0.46 (0.23–0.93)^*^	96	0.66 (0.35–1.28)
No	62	1.00 (Ref)	62	1.00 (Ref)	62	1.00 (Ref)	62	1.00 (Ref)
								
*Bilateral IFL*								
Yes	134	1.94 (0.54–6.92)	134	0.76 (0.32–1.85)	134	1.54 (0.58–4.11)	134	1.15 (0.50–2.64)
No	26	1.00 (Ref)	27	1.00 (Ref)	27	1.00 (Ref)	27	1.00 (Ref)
								
*En bloc surgery*								
Yes	71	2.59 (1.14–5.89)^*^	71	2.61 (1.29–5.26)^*^	71	0.42 (0.21–0.88)^*^	71	1.27 (0.67–2.40)
No	89	1.00 (Ref)	90	1.00 (Ref)	90	1.00 (Ref)	89	1.00 (Ref)
								
*Ligation saphenous vein*								
Yes	30	1.84 (0.72–4.68)	30	0.85 (0.35–2.08)	30	1.04 (0.44–2.49)	30	1.53 (0.66–3.53)
No	127	1.00 (Ref)	127	1.00 (Ref)	127	1.00 (Ref)	127	1.00 (Ref)
								
Total nodes dissected (number)	160	0.98 (0.89–1.07)	161	0.97 (0.89–1.05)	161	1.03 (0.95–1.11)	161	1.01 (0.94–1.08)
Total positive nodes (number)	160	0.63 (0.35–1.15)	161	1.07 (0.78–1.48)	161	0.97 (0.69–1.36)	161	0.91 (0.67–1.23)
Extracapsular growth (number)	160	0.67 (0.27–1.62)	161	1.20 (0.84–1.73)	161	0.96 (0.63–1.44)	161	0.93 (0.65–1.33)
Duration drain *in situ* (days)	148	0.99 (0.89–1.09)	148	1.04 (0.96–1.12)	148	1.02 (0.94–1.11)	148	1.01 (0.93–1.08)
Drain production last day (10 ml)	121	1.01 (0.97–1.05)	121	1.04 (1.00–1.08)^*^	121	1.05 (1.01–1.09)^*^	121	1.11 (1.04–1.19)^*^
Total drain production (10 ml)	145	1.00 (1.00–1.00)	145	1.00 (1.00–1.01)	145	1.00 (1.00–1.01)	145	1.00 (1.00–1.01)^*^

Abbreviations: *N*=patients with valid observation; OR=odds ratio; CI=confidence interval; IFL=inguinofemoral lymphadenectomy; (10 ml)=odds ratio assessed per 10 ml increase of lymph fluid; Ref=reference; BMI=body mass index.

^*^P<0.05.

**Table 4:** Odds ratios with 95% confidence interval of patient characteristics, surgery and postoperative management variables for long-term complications using univariate logistic regression

	**Lymphoedema**		**Cellulitis/erysipelas**		**Any long-term complication**	
**Variables**	***N***	**OR (95% CI)**	***N***	**OR (95% CI)**	***N***	**OR (95% CI)**
Age (years)	160	0.95 (0.93–0.98)^*^	160	0.96 (0.94–0.99)^*^	160	0.94 (0.92–0.97)^*^
BMI (in kg m^–2^)	152	1.02 (0.95–1.09)	152	1.04 (0.97–1.11)	152	1.04 (0.97–1.12)
						
*Bilateral IFL*						
Yes	133	1.78 (0.76–4.17)	133	0.84 (0.36–1.99)	133	1.04 (0.44–2.46)
No	27	1.00 (Ref)	27	1.00 (Ref)	27	1.00 (Ref)
						
*En bloc surgery*						
Yes	70	1.09 (0.59–2.04)	70	0.93 (0.48–1.81)	70	0.75 (0.39–1.44)
No	90	1.00 (Ref)	90	1.00 (Ref)	90	1.00 (Ref)
						
*Ligation saphenous vein*						
Yes	30	1.58 (0.71–3.53)	30	1.12 (0.49–2.56)	30	1.49 (0.63–3.51)
No	126	1.00 (Ref)	126	1.00 (Ref)	126	1.00 (Ref)
						
Total nodes dissected (number)	160	0.95 (0.88–1.02)	160	0.94 (0.87–1.02)	160	0.92 (0.85–0.99)^*^
Total positive nodes (number)	160	1.09 (0.80–1.49)	160	0.86 (0.61–1.23)	160	0.91 (0.67–1.24)
Extracapsular growth (number)	160	0.95 (0.66–1.36)	160	0.74 (0.42–1.30)	160	0.87 (0.60–1.24)
Duration drain *in situ* (days)	147	1.00 (0.93–1.07)	147	0.93 (0.85–1.01)	147	0.97 (0.90–1.05)
Drain production last day (10 ml)	120	1.00 (0.96–1.03)	120	1.02 (0.99–1.06)	120	1.01 (0.97–1.05)
Total drain production (10 ml)	144	1.00 (1.00–1.00)	144	1.00 (1.00–1.00)	144	1.00 (1.00–1.00)
						
*Adjuvant radiotherapy*						
Yes	39	1.02 (0.49–2.09)	39	1.80 (0.86–3.79)	39	1.40 (0.65–3.03)
No	120	1.00 (Ref)	120	1.00 (Ref)	120	1.00 (Ref)
						
*Wound breakdown*						
Yes	30	0.92 (0.41–2.03)	30	0.53 (0.21–1.33)	30	0.70 (0.31–1.57)
No	129	1.00 (Ref)	129	1.00 (Ref)	129	1.00 (Ref)
						
*Wound infection*						
Yes	46	0.84 (0.42–1.67)	49	0.93 (0.45–1.93)	49	0.96 (0.47–1.95)
No	114	1.00 (Ref)	114	1.00 (Ref)	114	1.00 (Ref)
						
*Lymphocele*						
Yes	47	0.62 (0.31–1.24)	47	2.54 (1.25–5.13)^*^	47	1.01 (0.50–2.04)
No	113	1.00 (Ref)	113	1.00 (Ref)	113	1.00 (Ref)
						
*Any short-term complication*						
Yes	94	0.67 (0.36–1.27)	94	1.66 (0.84–3.28)	94	0.90 (0.47–1.74)
No	65	1.00 (Ref)	65	1.00 (Ref)	65	1.00 (Ref)

Abbreviations: *N*=patients with valid observation; OR=odds ratio; CI=confidence interval; BMI=body mass index; IFL=inguinofemoral lymphadenectomy; (10 ml)=odds ratio assessed per 10 ml increase of lymph fluid; Ref=reference.

^*^P<0.05.

**Table 5:** Adjusted odds ratio with 95% confidence interval of patient characteristics, surgery and postoperative management variables for short- and long-term complications using multivariate logistic regression with selection procedure

	**Wound breakdown *N*=160**	**Wound infection *N*=121**	**Lymphocele *N*=121**	**Any short-term complication *N*=115**
**Short-term complications**	**OR (95% CI)**	**OR (95% CI)**	**OR (95% CI)**	**OR (95% CI)**
Age (years)	1.06 (1.02–1.10)	—	—	—
Diabetes	—	—	—	4.10 (1.05–16.05)
BMI	—	—	—	—
Continuation of antibiotics	—	—	—	—
En bloc surgery	2.72 (1.16–6.37)	2.66 (1.15–6.15)	—	—
Drain production last day (10 ml)	—	1.05 (1.00–1.09)	1.05 (1.01–1.10)	1.11 (1.04–1.19)
Total drain production (10 ml)	—	—	—	—
				
	**Lymphoedema**	**Cellulitis/erysipelas**		**Any long-term complication**
**Long-term complications**	**OR (95% CI)**	**OR (95% CI)**		**OR (95% CI)**
Age (years)	0.95 (0.93–0.98)	0.96 (0.93–0.98)		0.94 (0.92–0.97)
Total nodes dissected (number)	—	—		0.92 (0.84–1.00)
Duration drain *in situ* (days)	—	—	—	
Lymphocele	—	3.28 (1.50–7.19)	—	

Abbreviations: *N*=patients with valid observation; CI=confidence interval; OR=odds ratio; BMI=body mass index; —=not selected; (10 ml)=odds ratio assessed per 10 ml increase of lymph fluid.

**Table 6:** Literature overview of short- and long-term complication rate (%) in patients with vulvar SCC who underwent inguinofemoral lymphadenectomy

**Author**	**Year**	***N***	**Incision**	**After SLN**	**Wound breakdown**	**Wound infection**	**Lymphocele**	**Lymphoedema**	**Cellulitis/ erysipelas**
Podratz *et al*	1983	175	‘En bloc’	No	85 (with infection, necrosis)	85 (with breakdown and necrosis)	11	69	13 (with lymphangitis and phlebitis)
Hacker *et al*	1981	100	‘En bloc’	No	44	9	13	20	2
Gould *et al*	2001	67	Separate	No	23.6	35.4	18.1	34.3	22.2
Gaarenstroom *et al*	2003	101	Separate	No	17	39	40	28	—
van der Zee *et al*	2008	47 (short term) 119 (long term)	Separate	Yes	34	21.3	—	25.2	16.2
Hinten *et al*	2011	164	‘En bloc’ and separate	Yes	18.8	28.6	29.2	48.8	33.8

Abbreviations: SCC=squamous cell carcinoma; SLN=sentinel lymph node; *N*=number of patients; short term=wound breakdown and infection and lymphocele; long term=lymphoedema and cellulitis/erysipelas; —= not studied.
